# Supplementary material for: Characterization of HIV-1 Nucleoside-Modified mRNA Vaccines in Rabbits and Rhesus Macaques
Source: Mol Ther Nucleic Acids. 2019 Mar 21;15:36–47. doi: 10.1016/j.omtn.2019.03.003 (PMC6454128; doi:10.1016/j.omtn.2019.03.003)
Supplement: Document S1. Figures S1 and S2 and Table S1 [file mmc1.pdf]

OMTN, Volume *15*

Supplemental Information

Characterization of HIV-1 Nucleoside-Modified mRNA Vaccines in Rabbits and Rhesus Macaques

Norbert Pardi, Celia C. LaBranche, Guido Ferrari, Derek W. Cain, István Tombácz, Robert J. Parks, Hiromi Muramatsu, Barbara L. Mui, Ying K. Tam, Katalin Karikó, Patricia Polacino, Christopher J. Barbosa, Thomas D. Madden, Michael J. Hope, Barton F. Haynes, David C. Montefiori, Shiu-Lok Hu, and Drew Weissman

SUPPLEMENTAL FIGURES AND TABLES

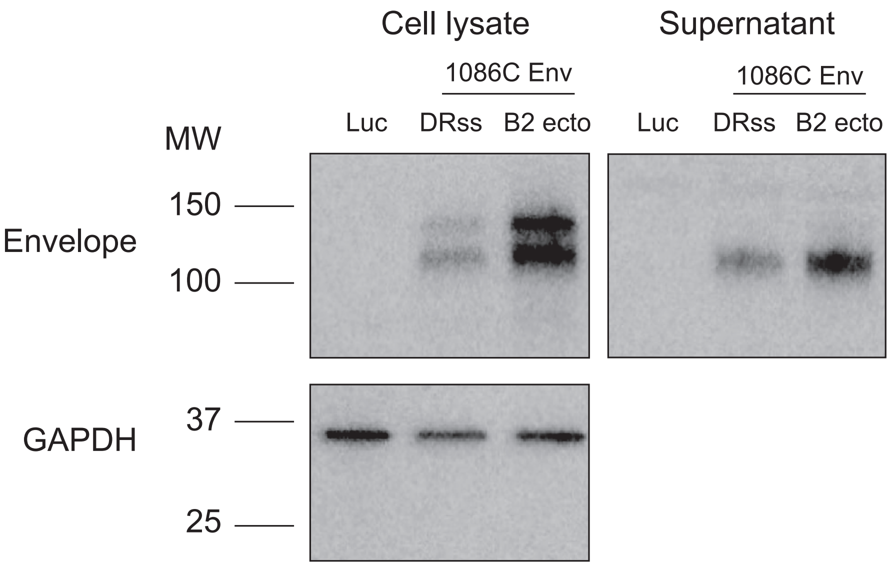

Figure S1. Protein production from HIV-1 1086C Env-encoding mRNAs in vitro
mRNAs were transfected into HEK293T cells. Env protein expression in cell lysate and supernatant was probed by Western blot, using firefly luciferase (Luc)-encoding mRNA-transfected cells as a negative control.

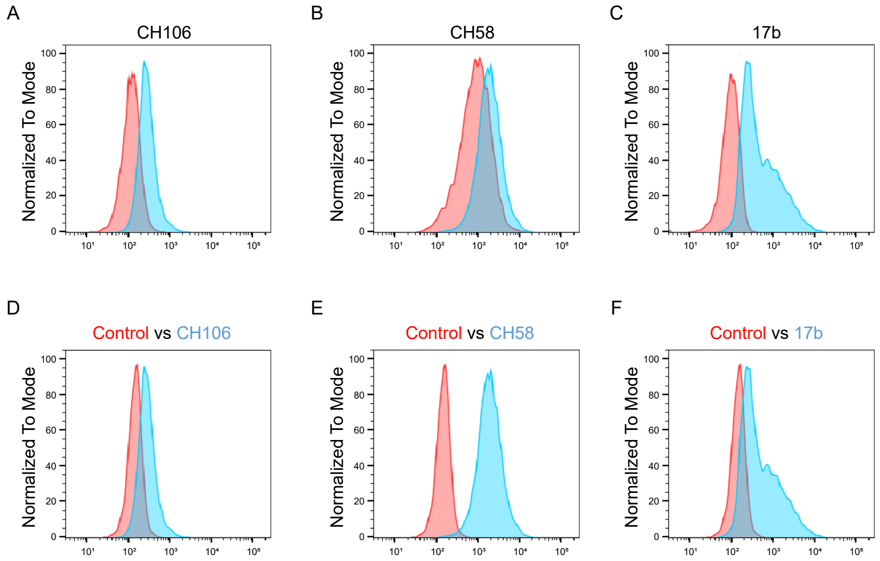

Figure S2. HIV nAb binding to 1086C B2 Ecto gp160 mRNA-transfected 293F cells
mRNAs were transfected into 293F cells. Panels A, B and C represent positive binding of three HIV gp120 antibodies to gp160 transfected cells (blue) relative to firefly luciferase transfected cells (red). Panels D, E and F show binding of the same three antibodies (blue) relative to an influenza virus anti-hemagglutinin negative control antibody, CH65 (red). Two independent experiments were performed with similar results.

ID	Sex	DOB	Age (years) Week 0	Weight (kg) Week 0
**A16331**	**Male**	**5/8/2013**	**3.81**	**4.07**
**A16332**	**Male**	**5/25/2013**	**3.77**	**4.48**
**A16333**	**Male**	**6/10/2013**	**3.72**	**4.67**
**A16334**	**Male**	**5/18/2013**	**3.79**	**3.83**
**A16335**	**Male**	**7/21/2013**	**3.61**	**6.48**
**A16337**	**Male**	**9/28/2013**	**3.42**	**4.20**

Table S1. Characteristics of rhesus macaques in vaccination experiments. Animal identification (ID) numbers, weight at the time of the first immunization, sex, and date of birth (DOB) are shown.
