# Supplementary material for: Characterization of HIV-1 Nucleoside-Modified mRNA Vaccines in Rabbits and Rhesus Macaques
Source: Mol Ther Nucleic Acids. 2019 Mar 21;15:36–47. doi: 10.1016/j.omtn.2019.03.003 (PMC6454128; doi:10.1016/j.omtn.2019.03.003)
Supplement: Document S2. Article plus Supplemental Information [file mmc2.pdf]

Molecular Therapy
Nucleic Acids
Original Article

AMERICAN SOCIETY of GENE & CELL THERAPY

Characterization of HIV-1 Nucleoside-Modified mRNA Vaccines in Rabbits and Rhesus Macaques

Norbert Pardi,[1] Celia C. LaBranche,[2] Guido Ferrari,[2] Derek W. Cain,[3] István Tombácz,[1] Robert J. Parks,[3] Hiromi Muramatsu,[1] Barbara L. Mui,[4] Ying K. Tam,[4] Katalin Karikó,[5] Patricia Polacino,[6] Christopher J. Barbosa,[4] Thomas D. Madden,[4] Michael J. Hope,[4] Barton F. Haynes,[3] David C. Montefiori,[2] Shiu-Lok Hu,[6,7] and Drew Weissman[1]

[1]Department of Medicine, University of Pennsylvania, Philadelphia, PA 19104, USA; [2]Department of Surgery, Duke University Medical Center, Durham, NC 27710, USA; [3]Duke Human Vaccine Institute, Duke University School of Medicine, Durham, NC 27710, USA; [4]Acuitas Therapeutics, Vancouver, BC V6T 1Z3, Canada; [5]BioNTech RNA Pharmaceuticals, An der Goldgrube 12, 55131 Mainz, Germany; [6]Washington National Primate Research Center, University of Washington, Seattle, WA 98195, USA; [7]Department of Pharmaceutics, University of Washington, Seattle, WA 98195, USA

Despite the enormous effort in the development of effective vaccines against HIV-1, no vaccine candidate has elicited broadly neutralizing antibodies in humans. Thus, generation of more effective anti-HIV vaccines is critically needed. Here we characterize the immune responses induced by nucleoside-modified and purified mRNA-lipid nanoparticle (mRNA-LNP) vaccines encoding the clade C transmitted/founder HIV-1 envelope (Env) 1086C. Intradermal vaccination with nucleoside-modified 1086C Env mRNA-LNPs elicited high levels of gp120-specific antibodies in rabbits and rhesus macaques. Antibodies generated in rabbits neutralized a tier 1 virus, but no tier 2 neutralization activity could be measured. Importantly, three of six non-human primates developed antibodies that neutralized the autologous tier 2 strain. Despite stable anti-gp120 immunoglobulin G (IgG) levels, tier 2 neutralization titers started to drop 4 weeks after booster immunizations. Serum from both immunized rabbits and non-human primates demonstrated antibody-dependent cellular cytotoxicity activity. Collectively, these results are supportive of continued development of nucleoside-modified and purified mRNA-LNP vaccines for HIV. Optimization of Env immunogens and vaccination protocols are needed to increase antibody neutralization breadth and durability.

INTRODUCTION

There has been great progress in understanding the biology of human immunodeficiency virus type 1 (HIV-1) infection, but no effective vaccine has advanced to clinical development. Generation of broadly neutralizing antibodies (bnAbs) that recognize a wide range of HIV-1 isolates has been difficult due to the large number of host glycans that are linked to the HIV-1 envelope (Env), the rapid mutability of viral immunogens, and the ability of the virus to hide neutralization epitopes.[1] Interestingly, some HIV-infected individuals develop bnAbs years after natural infection, which suggests that generation of antibody-based protective vaccines may be possible. The tremendous efforts to elicit potent bnAbs against HIV-1 in small and large animals using various vaccine platforms (protein, viral vectors, DNA) and optimized Env immunogens[2,3] have largely failed; thus, the development of new vaccine types, immunization schemes, and vaccine immunogens remains a global priority.

Lipid nanoparticle (LNP)-encapsulated nucleoside-modified mRNA vaccines have recently demonstrated protective efficacy against various viral pathogens in preclinical studies.[4–9] We are just beginning to learn the mechanisms of action of nucleoside-modified mRNA-LNP vaccines, and they appear to have features that are found to be important for the development of bnAbs against HIV-1. Most notably, recent studies have demonstrated that this vaccine type has the ability to efficiently activate T follicular helper (Tfh) cells[6,10] that drive germinal center (GC) reactions, leading to durable, high-affinity neutralizing antibody responses.[11] Generation of HIV bnAbs in infected patients can take 2–4 years due to their complexity (multiple somatic hypermutations, long complementarity determining regions [CDRs]); thus, it has been widely accepted that strong and sustained GC reactions are prerequisites for the production of such antibodies.[12]

Here we report on studies where rabbits and rhesus macaques were immunized with nucleoside-modified mRNA-LNPs encoding the clade C transmitted/founder 1086C HIV-1 Env.[13] mRNA constructs induced high titers of anti-gp120 antibodies, as well as antibody-dependent cellular cytotoxicity activity in rabbits and non-human primates. Importantly, 3 of 6 rhesus monkeys developed neutralizing antibodies against the autologous tier 2 strain after 3 or 4 immunizations.

Received 4 February 2019; accepted 12 March 2019;
https://doi.org/10.1016/j.omtn.2019.03.003.

Correspondence: Norbert Pardi, Department of Medicine, University of Pennsylvania, Philadelphia, PA 19104, USA.
E-mail: pnorbert@pennmedicine.upenn.edu
Correspondence: Drew Weissman, Department of Medicine, University of Pennsylvania, Philadelphia, PA 19104, USA.
E-mail: dreww@pennmedicine.upenn.edu

This is an open access article under the CC BY license (http://creativecommons.org/licenses/by/4.0/).

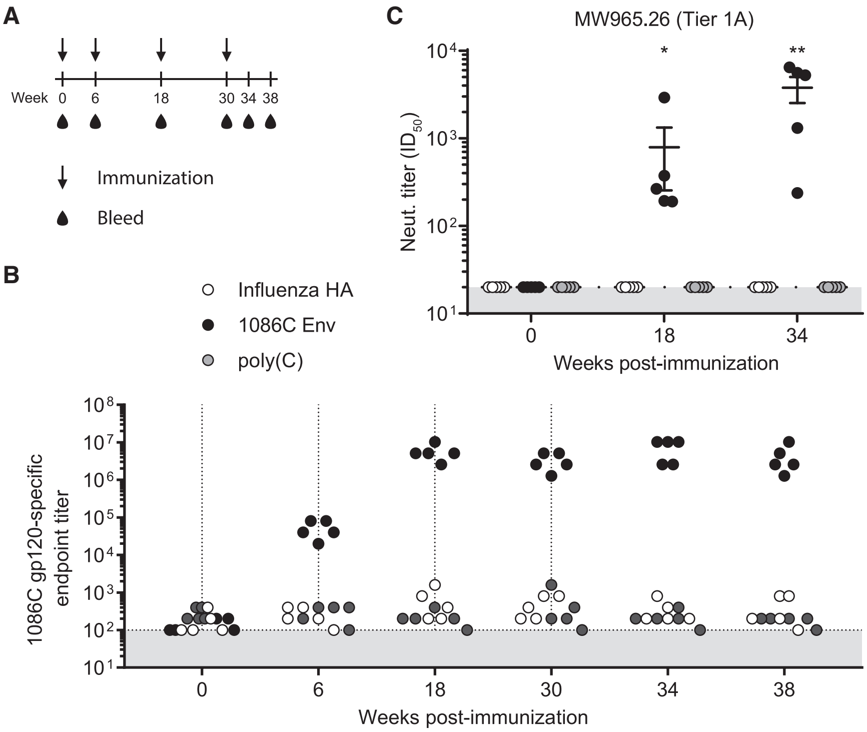

Figure 1. Antibody Responses after Nucleoside-Modified 1086C Env mRNA-LNP Immunization in Rabbits

(A) Schematic of experimental design. Rabbits were immunized intradermally with 50 μg HIV-1 1086C B2 ecto Env or influenza virus A/California/07/2009 HA-encoding nucleoside-modified mRNA-LNPs or poly(C) RNA-LNPs at weeks 0, 6, 18, and 30. (B) Sera were collected at weeks 0, 6, 18, 30, 34, and 38, and the kinetics of 1086C anti-gp120 IgG titers were determined by endpoint dilution ELISA. Vertical black arrows indicate dates of immunizations. Shaded area indicates the limit of detection. n = 5 rabbits and each symbol represents one animal. (C) Neutralization titers (expressed as the reciprocal serum dilution resulting in 50% inhibition of infection) from sera were determined against the MW965.26 (tier 1A) virus. Each symbol represents one animal. Means of values with SEM are displayed. Statistical significance of differences (denoted by asterisks) was determined using two-way ANOVA with Bonferroni multiple comparisons after log transformation of values; $p < 0.0001$. ** denotes significant difference to *$p < 0.005$.

RESULTS

In Vitro Characterization of Nucleoside-Modified 1086C Env mRNA Vaccine Immunogens

Protein production from mRNAs encoding 1086C.DRss and 1086C B2 ecto Env gp160s was confirmed by western blot analyses on cell lysates obtained from Env mRNA-transfected HEK293T cells (Figure S1). The DRss and B2 ecto Env constructs differed in their signal sequences and several residues in their cytoplasmic tails.

Next, the ability of selected HIV-1 anti-Env antibodies to bind to 1086C Env protein expressed on the surface of transfected 293F cells was determined by flow cytometry. 1086C Env gp160 binding to antibodies CH58,[14] CH106,[15] and 17b[16] was observed (Figure S2). These data suggested that the nucleoside-modified mRNA-LNP-encoded 1086C Env gp160 expressed on the 293F cell surface is capable of presenting antibody binding to V2 (CH58), CD4 (CH106), and a CD4-inducible (17b) epitope.

Immunization with Nucleoside-Modified 1086C Env mRNA-LNPs Elicits High Levels of Antigen-Specific Antibodies with ADCC and Tier 1 Virus Neutralization Activity in Rabbits

New Zealand white rabbits were intradermally immunized with 50 μg (~0.025 mg/kg) 1086C B2 ecto Env, influenza virus A/California/07/2009 hemagglutinin (HA)-encoding[8] purified, nucleoside-modified mRNA-LNPs, or poly(C) RNA-LNPs at weeks 0, 6, 18, and 30 (Figure 1A), and 1086C anti-gp120 immunoglobulin G (IgG) titers were determined at weeks 0, 6, 18, 30, 34, and 38 sera by ELISA. Env mRNA-LNP-vaccinated animals developed antigen-specific antibodies 6 weeks after a single immunization (Figure 1B). Administration of subsequent doses potently boosted anti-gp120-specific antibody titers that remained stable over the course of the study (Figure 1B).

Next, HIV-1 pseudovirus neutralization assays were performed on pre-immune and week 18 and 34 sera using the standard TZM-bl luciferase reporter system.[17] A murine leukemia virus (MLV) Env-expressing pseudovirus was used as a negative control. All Env mRNA-LNP-vaccinated animals developed neutralizing antibodies against the tier 1A virus, MW965.26, after 2 immunizations (Figures 1C and 2). Significantly increased neutralization activity was measured in week 34 samples. No tier 2 neutralization activity could be measured in immunized rabbits (Figure 2).

The effector function of non-neutralizing antibody responses is often measured by antibody-dependent cellular cytotoxicity (ADCC) assays. Importantly, studies investigating antibody responses from the RV144 trial suggest that ADCC likely contributed to vaccine efficacy.[18] To measure ADCC activity in rabbits, we utilized two approaches: the flow cytometry-based GranToxiLux (GTL) assay that measures responses against 1086C gp120-coated target cells and a luciferase-based assay that measures ADCC activity against HIV-1 1086C transmitted/founder infectious molecular clone (IMC)-infected cells. Both assays revealed that all animals immunized with Env mRNA-LNPs developed significant ADCC responses after 2 immunizations (week 18) that were further increased after 4 immunizations (week 34) (Figure 3).

Group	Animal ID	Neut. titer (ID_{50})											
		SVA-MLV			MW965.26			Ce1086_B2			Ce1176_A3		
		Negative Control			Tier 1A, Clade C			Tier 2, Clade C			Tier 2, Clade C		
		w0	w18	w34	w0	w18	w34	w0	w18	w34	w0	w18	w34
Influenza HA	27589	<20	<20	<20	<20	<20	<20	<20	<20	<20	<20	<20	<20
	27590	22	<20	<20	<20	<20	<20	<20	<20	<20	<20	<20	<20
	27591	<20	<20	<20	<20	<20	<20	<20	<20	<20	<20	<20	<20
	27592	<20	<20	<20	<20	<20	<20	<20	<20	<20	<20	<20	<20
	27594	<20	<20	<20	<20	<20	<20	<20	<20	<20	<20	<20	<20
1086C Env	27595	<20	<20	<20	<20	266	238	<20	<20	<20	<20	<20	<20
	27596	<20	<20	<20	<20	2933	6486	<20	<20	<20	<20	<20	<20
	27597	<20	<20	<20	<20	190	5260	<20	<20	<20	<20	<20	<20
	27599	<20	<20	<20	<20	193	1313	<20	<20	<20	<20	<20	<20
	27600	<20	<20	<20	<20	375	5622	<20	<20	<20	<20	<20	<20
poly(C)	27606	<20	<20	<20	<20	<20	<20	<20	<20	<20	<20	<20	<20
	27607	<20	<20	<20	<20	<20	<20	<20	<20	<20	<20	<20	<20
	27608	<20	<20	<20	<20	<20	<20	<20	<20	<20	<20	<20	<20
	27609	<20	<20	<20	<20	<20	<20	<20	<20	<20	<20	<20	<20
	27610	<20	<20	<20	<20	<20	<20	<20	<20	<20	<20	<20	<20

Figure 2. Neutralizing Antibody Titers in Rabbits

Neutralizing antibodies generated by 1086C B2 ecto Env mRNA-LNP immunizations in rabbits were measured in week 0, 18, and 34 sera by the TZM-bl assay against HIV-1 isolates MW965.26 (tier 1A), Ce1086_B2 (autologous tier 2), and Ce1176_A3 (tier 2). Data are reported as dilution of sera required to inhibit 50% of viral infectivity (ID_{50}) and labeled in blue (21–100), yellow (101–1,000), orange (1,001–5,000), and red (>5,001).

Immunization with Nucleoside-Modified 1086C Env mRNA-LNPs Elicits High Levels of Antigen-Specific Antibodies with ADCC and HIV-1 Neutralization Activity in Rhesus Macaques

Rhesus macaques (Table S1) were intradermally immunized with 50 μg (~0.014 mg/kg) 1086C.DRss Env mRNA-LNPs at weeks 0, 4, 20, 32, and 48 (Figure 4A). First, 1086C Env gp120-specific IgG titers were determined from serum samples collected at weeks 0, 4, 8, 24, 34, 40, 50, and 52. A single immunization elicited anti-gp120 IgG responses that were potently boosted by subsequent injections. All animals developed high levels of anti-gp120 IgG by week 8 that remained relatively stable over the course of the study (Figure 4B). Of note, the pre-immune sample from one animal had unusually high gp120-specific binding activity.

Next, monkey sera were examined by HIV-1 pseudovirus neutralization assays.[17] MLV Env-expressing pseudovirus was used as a negative control. All animals developed antibodies that neutralized the tier 1A virus MW965.26 after 2 immunizations (Figures 4C and 5). Significantly increased tier 1A virus neutralization activity was measured after booster immunizations in samples taken at weeks 24, 34, 40, 50, and 52. Of note, serum neutralization titers started to drop 4–6 weeks after booster immunizations, as can be seen between weeks 34 and 40 or between weeks 50 and 52. Five of 6 animals developed low but measurable serum neutralization activity against a clade C tier 1B virus (92BR025.9) after 5 immunizations (Figures 4D and 5). Most importantly, 3 of 6 monkeys generated neutralizing antibodies against the autologous tier 2 1086C virus after 3 or 4 immunizations (Figures 4E and 5). This neutralization activity was low and waned shortly after booster vaccinations in 2 animals, but, interestingly, animal A16334 developed extremely high and relatively stable tier 2 virus neutralization titers. Finally, low and transient heterologous tier 2 virus (25710-2.43 and Ce1176_A3) neutralization titers were measured in animal A16337 (Figure 5).

As animal A16334 displayed potent autologous tier 2 virus neutralization activity, pseudovirus neutralization epitope-mapping assays were performed by using a panel of mapping mutants of 1086C Env that eliminate or introduce the binding sites of the known classes of bnAbs (Figure 6). Serum from week 34 was assayed against this panel of viruses, and several bnAbs were included to document the phenotype of the mutant viruses assayed.[19–24] Similar to the previous studies, potent neutralization of the parent 1086_B2 virus was detected, and no decrease was observed for the CD4-binding site mutants N276Q, N279Q, and N280D. N332A, which targets the V3 glycan-dependent epitope, maintained neutralization. For gp120-gp41 interface mutations N88A and N625A, a small decrease in neutralization was observed. V295N designed to re-introduce the 2G12 epitope had no effect on neutralization. Interestingly, neutralization was eliminated by the K160N mutation, which introduces the V2 glycan-dependent bnAb epitope that is missing in the 1086C Env. This mutation restores neutralization by the bnAbs PG9, PG16, CH01, and PGDM1400, but it completely blocks neutralization by serum from animal A16334.

This loss of neutralization by the addition of a glycan could be similar in mechanism to the filling of glycan holes previously described for autologous neutralization of BG505 Env.[25,26] Surprisingly, neutralization by the A16334 serum was also eliminated by the N611A

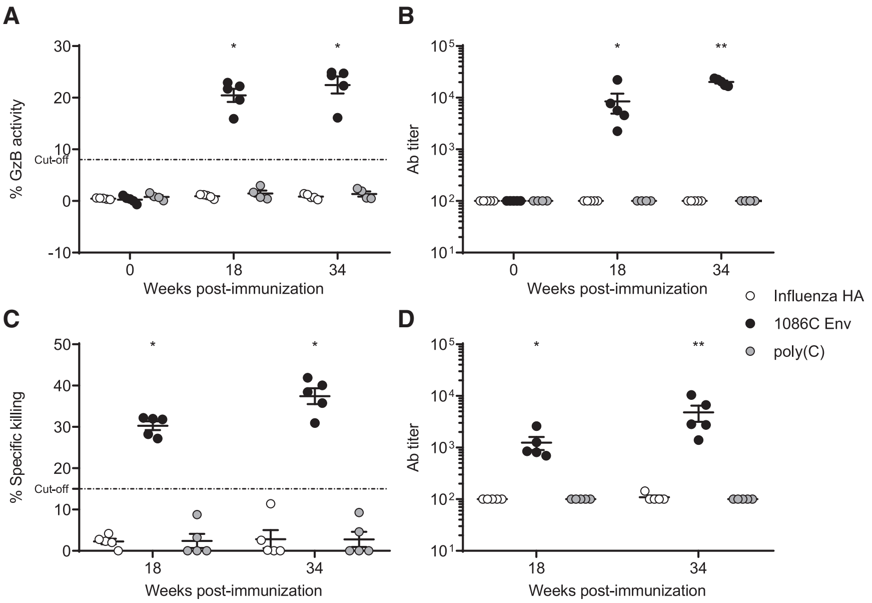

Figure 3. ADCC Activity after Nucleoside-Modified 1086C Env mRNA-LNP Immunization in Rabbits

ADCC activity in rabbit sera obtained at weeks 0, 18, and 34 was assessed. (A and B) ADCC responses against the 1086C recombinant gp120-coated target cells (GTL assay). Granzyme B activity (A) and ADCC antibody titers (B) were determined. The cut-off for positivity (indicated with a horizontal dotted line) was $\geq$8% of granzyme B activity after subtracting the background measured in the uncoated cells. Antibody titers are expressed as the dilution at which the dilution curve interpolate with the 15% cut-off value. Each symbol represents one animal. (C and D) ADCC responses against HIV-infected cells (luciferase-based assay). Specific killing activity (C) and ADCC antibody titers (D) were determined. The analysis of the results was conducted after subtracting the background detected with the pre-immunization (week 0) samples. The horizontal dotted line indicates the cut-off for positivity. n = 5 rabbits and each symbol represents one animal. Means of values with SEM are displayed. Statistical significance of differences (denoted by asterisks) was determined using two-way ANOVA with Bonferroni multiple comparisons after log transformation of values; $p < 0.0001$. ** denotes significant difference to *$p < 0.005$.

mutation. As noted in the figure legend, there was still positive deflection in the neutralization curve that reached a maximum of 44% for the serum, but neutralization by serum was substantially reduced by removal of the N611 glycan. The N611A mutation has been shown to reduce or abrogate neutralization of the gp120-41 interface bnAb PGT151 in most HIV strains.[20] In contrast, Ce1086_B2.N611A is more sensitive to neutralization by PGT151 than the parent virus, and, surprisingly, N611A also enables neutralization by the V2 glycan-dependent bnAbs PG9, PG16, PGDM1400, and CH01. We have sequenced the Env expression plasmid used to make this Ce1086_B2.N611A pseudovirus, we have confirmed the presence of both K160 and the N611A mutation, and we have repeated neutralization assays of Ce1086_B2.N611A with PGT151 and the V2 glycan-dependent bnAbs several times with the same results (data not shown).

ADCC activity in non-human primate (NHP) week 34 sera was measured using the GTL and luciferase-based assays (Figure 7). ADCC responses against gp120-coated target cells were detected against both the subtype C HIV-1 TV-1 and 1086C gp120-coated target cells in 5 of 6 animals (A16337 was a non-responder) (Figure 7A). The mean of the maximum granzyme B activity was similar against TV-1 (11.88 ± 3.59) compared to 1086C (11.71 ± 3.76). The mean antibody titers were higher against 1086C (1:10,891 ± 11,520) compared to TV-1 (1:2,774 ± 2,379) (Figure 7B). Interestingly, ADCC responses against either the TV-1 or 1086C IMC-infected cells were not detectable in week 34 NHP sera.

DISCUSSION

HIV-1 infection is a global health threat with approximately 2 million new adult infections every year. Although the widespread use of antiretroviral drugs has been very effective in controlling the virus in infected individuals and decreasing the probability of spreading the infection, generation of an effective anti-HIV vaccine is likely to be necessary to end the pandemic. Since bnAbs have the ability to neutralize multiple circulating strains of HIV-1, they are in the forefront of vaccine development. Unfortunately, generation of potent bnAbs has been extremely difficult, and it has been shown to occur only in a fraction of HIV-infected individuals years after natural infection.[27] The production of bnAbs has never been achieved by any HIV vaccine in humans.

The disappointing results from multiple preclinical and several clinical HIV vaccine studies have made it clear that traditional vaccine approaches will not be sufficient to elicit broadly protective anti-HIV immune responses and that the rational design of new immunization schemes, vaccine modalities, and immunogens is necessary to create an effective antibody-driven vaccine. Rational vaccine design will likely benefit from better understanding the biology of HIV infection, investigating the structure of bnAbs, and identifying elements of the immune system that are necessary for bnAb production. Multiple studies revealed that bnAbs have unusually high numbers of somatic hypermutations, including rare insertions, deletions, and cys-cys double mutations that are often critical for the bnAb activity.[28–33] Generation of somatic mutations (antibody affinity maturation) occurs in the GCs through the dynamic interactions of Tfh and GC B cells.[11] A positive correlation between the frequencies of antigen-specific Tfh cells and induction of neutralizing antibody breadth has been demonstrated in simian immunodeficiency virus (SIV) and simian/human immunodeficiency virus (SHIV) infection studies in non-human primates.[34–38] Therefore, identification of new adjuvants and vaccine platforms that promote sustained GC reactions through the potent activation of Tfh and GC B cells is critically important for the

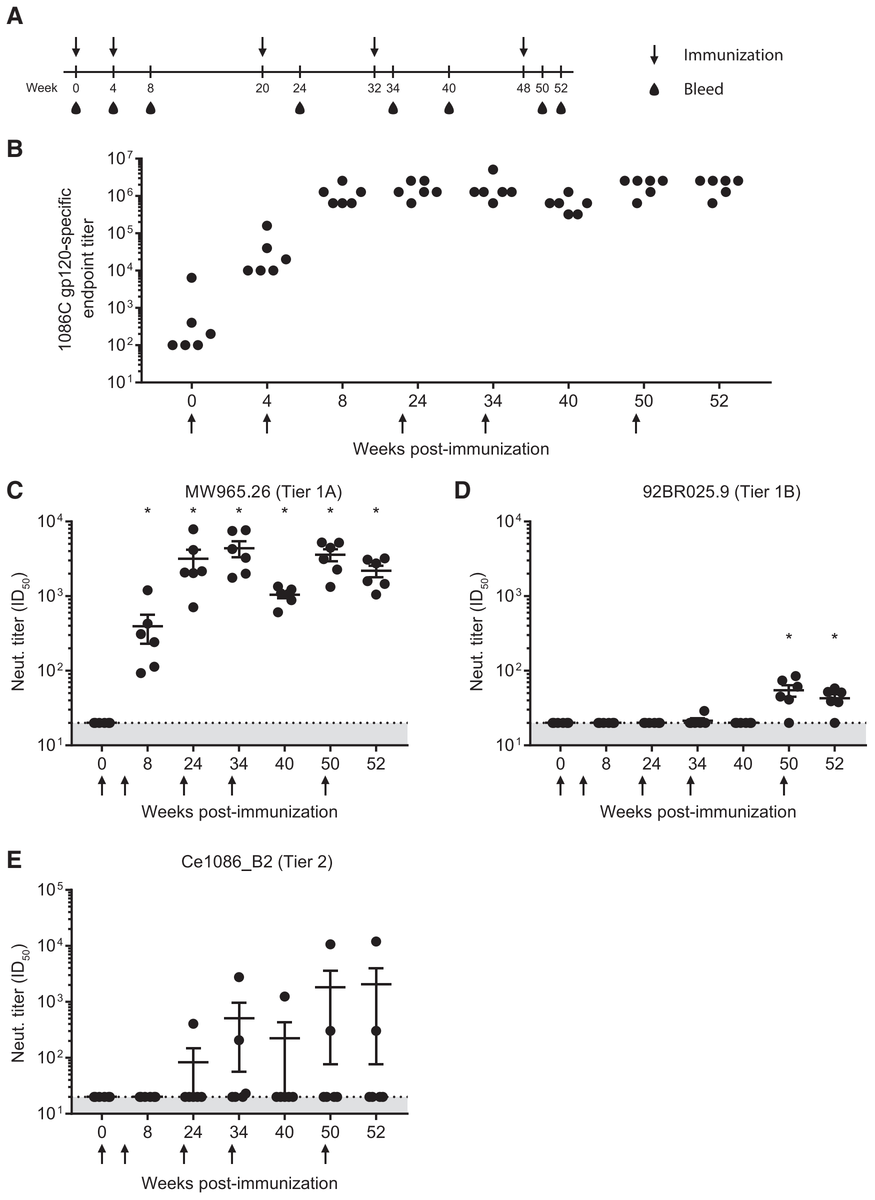

Figure 4. Antibody Responses after Nucleoside-Modified 1086C Env mRNA-LNP Immunization in Rhesus Macaques

(A) Schematic of experimental design. Rhesus macaques were immunized intradermally with 50 μg HIV-1 1086C.DRss Env mRNA-LNPs at weeks 0, 4, 20, 32, and 48. (B) Sera were collected at weeks 0, 4, 8, 24, 34, 40, 50, and 52, and the kinetics of 1086C anti-gp120 IgG titers were determined by endpoint dilution ELISA. Vertical black arrows indicate dates of immunizations. Shaded area indicates the limit of detection. Each symbol represents one animal. (C–E) Neutralization titers (expressed as the reciprocal serum dilution resulting in 50% inhibition of infection) from sera were determined against MW965.26 (C, tier 1A), 92BR025.9 (D, tier 1B), and Ce1086_B2 (E, autologous tier 2). n = 6 monkeys and each symbol represents one animal. Means of values with SEM are displayed. Statistical significance of differences (denoted by asterisk) was determined using one-way ANOVA with Bonferroni multiple comparisons to week 0 samples after log transformation of values; $p < 0.0001$.

development of an effective, broadly protective antibody-driven HIV vaccine.

It has recently been demonstrated that nucleoside-modified and purified mRNA-LNP vaccines have the ability to induce strong GC Tfh cell responses and durable neutralizing antibody responses in NHPs.[6] In this study, we show that 3 of 6 rhesus macaques, immunized with HIV-1 1086C Env gp160 nucleoside-modified mRNA-LNP vaccine, generated antibodies with autologous tier 2 neutralization activity and one animal developed short-lived antibodies with weak heterologous tier 2 virus neutralization ability (Figures 4 and 5). Interestingly, despite the relatively stable and high Env-specific IgG titers in all monkeys, both the tier 1 and tier 2 virus neutralization activities of Env-specific antibodies started to decline 4 weeks after each immunization (Figures 4B and 5). Of note, one animal displayed high anti-gp120-specific IgG responses in pre-immunization serum that could be due to cross-reactive anti-HIV responses with commensal, environmental, or even self antigens, as reported by several studies.[39–41]

To gain more insights into the specificity of the tier 2 antibodies elicited by 1086C Env mRNA-LNP immunizations, epitope-mapping experiments were performed using pseudoviruses with resistance mutations for known bnAbs. Since animal A16334 developed extremely high autologous tier 2 virus-neutralizing responses, we tested week 34 serum from this animal. These studies revealed that antibodies developed by this rhesus monkey likely target a glycan hole in the vicinity of the V2 loop, as neutralization was abrogated by adding a glycan residue with the K160N mutation. Interestingly, the N611A mutation in gp41 also eliminated neutralization by serum from A16334. This mutation reduces neutralization by PGT151 in most HIV strains, but our data show that N611A actually increases the sensitivity of 1086C to PGT151 and also enables neutralization of this mutant virus by V2 glycan-dependent bnAbs. We believe that there are two possible explanations for the increased V2 glycan binding: either by rearrangement of apex and other glycans or by gp41-induced conformational changes.[42–45]

As a positive correlation was demonstrated between the ADCC activity of antibodies and vaccine efficacy in the RV144 trial,[18] ADCC

		Neut. titer (ID50)					
		SVA-MLV	MW965.26	92BR025.9	Ce1086_B2	25710-2.43	Ce1176_A3
		Negative Control	Clade C	Clade C	Clade C	Clade C	Clade C
Animal ID	Bleed Week		Tier 1A	Tier 1B	Tier 2	Tier 2	Tier 2
A16331	0	<20	<20	<20	<20	<20	<20
	8	<20	**242**	<20	<20	<20	<20
	24	<20	**2032**	<20	<20	<20	<20
	34	<20	**4274**	<20	<20	<20	<20
	40	<20	**1072**	<20	<20	<20	<20
	50	<20	**5245**	**74**	<20	<20	<20
	52	<20	**3081**	**51**	<20	<20	<20
A16332	0	<20	<20	<20	<20	<20	<20
	8	<20	**1202**	<20	<20	<20	<20
	24	<20	**7854**	<20	<20	<20	<20
	34	<20	**7644**	<20	<20	<20	<20
	40	<20	**1225**	<20	<20	<20	<20
	50	<20	**4447**	<20	<20	<20	<20
	52	<20	**3206**	<20	<20	<20	<20
A16333	0	<20	<20	<20	<20	<20	<20
	8	<20	**310**	<20	<20	<20	<20
	24	<20	**2152**	<20	<20	<20	<20
	34	<20	**1998**	<20	<20	<20	<20
	40	<20	**609**	<20	<20	<20	<20
	50	<20	**3138**	**85**	<20	<20	<20
	52	<20	**1460**	**58**	<20	<20	<20
A16334	0	<20	<20	<20	<20	<20	<20
	8	<20	**427**	<20	<20	<20	<20
	24	<20	**4161**	<20	**405**	<20	<20
	34	<20	**3270**	<20	**2761**	<20	<20
	40	<20	**1085**	<20	**1242**	<20	<20
	50	<20	**2265**	**41**	**10696**	<20	<20
	52	<20	**1589**	**38**	**11949**	<20	<20
A16335	0	<20	<20	<20	<20	<20	<20
	8	<20	**113**	<20	<20	<20	<20
	24	<20	**2068**	<20	<20	<20	<20
	34	<20	**7456**	<20	**23**	<20	<20
	40	<20	**1346**	<20	<20	<20	<20
	50	<20	**5207**	**61**	<20	<20	<20
	52	<20	**2747**	**39**	<20	<20	<20
A16337	0	<20	<20	<20	<20	<20	<20
	8	<20	**93**	<20	<20	<20	<20
	24	<20	**711**	<20	<20	<20	<20
	34	<20	**1758**	**29**	**207**	**21**	**31**
	40	<20	**885**	<20	<20	<20	<20
	50	<20	**1329**	**45**	**303**	<20	<20
	52	<20	**1050**	**52**	**303**	<20	<20

Figure 5. Neutralizing Antibody Titers in Rhesus Macaques

Neutralizing antibodies generated by 1086C.DRss Env mRNA-LNP immunizations in rhesus macaques were measured in week 0, 8, 24, 34, 40, 50, and 52 sera by the TZM-bl assay against HIV-1 isolates MW965.26 (tier 1A), 92BR025.9 (tier 1B), Ce1086_B2 (autologous tier 2), 25710-2.43 (tier 2), and Ce1176_A3 (tier 2). Data are reported as dilution of sera required to inhibit 50% of viral infectivity (ID_{50}) and labeled in blue (21–100), yellow (101–1,000), orange (1,001–5,000), and red (>5,001).

	Neutralization titer (ID_{50} for sera and IC_{50} for bnAbs)									
Recognition site:	Parent	V2 glycan	V3 glycan	2G12 sensitivity	CD4 Binding site			gp120-41 interface		
Mapping mutant:	-	K160N	N332A	V295N	N276Q	N279Q	N280D	N88A	N611A	N625A
A16334	3356	<20	2980	3476	3243	4246	3044	2453	*<20	2530
PG9 (μg/ml)	>10	0.027	>10	>10	>10	>10	>10	>10	0.032	>10
PG16 (μg/ml)	>10	<0.005	>10	>10	>10	>10	>10	>10	<0.005	>10
CH01 (μg/ml)	>10	1.323	>10	>10	>10	>10	>10	>10	9.005	>10
PGDM1400 (μg/ml)	>10	<0.005	>10	>10	>10	>10	>10	>10	0.022	>10
CH31 (μg/ml)	3.09	2.146	2.693	3.869	>10	>10	>10	2.621	0.064	2.479
VRC01 (μg/ml)	0.385	0.399	0.515	1.047	0.585	>10	>10	0.511	0.142	0.526
PGT151 (μg/ml)	1.918	0.109	1.007	4.901	0.15	0.43	0.184	1.853	0.005	1.933
VRC34.01 (μg/ml)	0.046	0.028	0.045	0.061	0.048	0.05	0.038	>10	0.024	0.015

Figure 6. Neutralizing Antibody Epitope Mapping in Rhesus Monkey A16334

Epitope-mapping experiments were performed with week 34 serum from rhesus macaque A16334 using pseudoviruses with resistance mutations for known bnAbs targeting various regions of the HIV-1 envelope. Concentration (μg/mL) of bnAbs required to inhibit 50% of viral infectivity (IC_{50}) is shown. Serum neutralization titers are reported as dilution of serum required to inhibit 50% of viral infectivity (ID_{50}). *Positive deflection (44%) was detected for Ce1086_B2.N611A.

assays were performed on rabbit and monkey samples. All rabbits and 5 of 6 1086C Env mRNA-LNP-immunized NHPs developed antibodies with ADCC activity (Figures 3 and 7). Interestingly, rabbit serum showed ADCC against both 1086C recombinant gp120-coated and virus-infected target cells (Figure 3), while no ADCC was detectable against infected cells in NHPs. Additionally, rabbit antibodies seemed to recruit human natural killer (NK) cells (used as effector cells in these assays) much better than the NHP antibodies (G.F., unpublished data).

To our knowledge, this is the first study that evaluates nucleoside-modified mRNA-LNP HIV vaccine efficacy in large animals. We demonstrate that nucleoside-modified mRNA-LNP vaccination induces high levels of antigen-specific IgG in both rhesus monkeys and rabbits. Interestingly, none of the rabbits and only half of the monkeys developed antibodies with tier 2 neutralization activity. Previous studies demonstrated that one or two immunizations with nucleoside-modified mRNA-LNPs induced complete and durable protection against various infectious diseases in large animals.[5,7] Here we found that five injections with HIV Env mRNA-LNP vaccines did not result in broad and durable neutralizing antibody responses, which further demonstrates the extreme difficulty of generating effective HIV vaccines. We vaccinated animals with wild-type HIV Env gp160-encoding mRNA constructs, which may not be the best immunogens for the induction of bnAb responses. We believe that the use of mRNA-encoded rationally designed next-generation immunogens, such as stabilized SOSIPs[46] or cell surface trimers[47] that better mimic bnAb epitopes and thus allow the development of Abs with greater potency and B cell lineage immunogens[48] that enable the generation of rare bnAbs through the stimulation of various bnAb clonal lineages, would be a promising future direction. It is also possible that immunization schemes need to be optimized: numerous studies[49] demonstrated that prime-boost immunizations (for example, DNA prime-viral vector boost or DNA prime-protein boost) led to more robust immune responses; thus, it would be intriguing to perform experiments that use mRNA vaccines for prime and protein vaccines for boost. These approaches may lead to more effective vaccines with increased neutralization breadth and durability, which would be worth evaluating in clinical trials.

MATERIALS AND METHODS

Ethics Statement

The investigators faithfully adhered to the "Guide for the Care and Use of Laboratory Animals" by the Committee on Care of Laboratory Animal Resources Commission on Life Sciences, National Research Council.

Rabbits

The animal facility at the University of Pennsylvania is fully accredited by the American Association for Accreditation of Laboratory Animal Care (AAALAC). All studies were conducted under protocols approved by the University of Pennsylvania Institutional Animal Care and Use Committee (IACUC).

Monkeys

Indian origin rhesus macaques (*Macaca mulatta*) were housed in AAALAC-accredited facilities, and all procedures were conducted with University of Washington IACUC approval.

mRNA Production

mRNAs were produced as previously described,[50] using T7 RNA polymerase (Megascript, Ambion) on linearized plasmids encoding codon-optimized[51] firefly luciferase (pTEV-new Luc2-A101), two variants of clade C 1086C Env[13] (pTEV-1086C.DRss Env-A101 and pTEV-1086C B2 ecto Env-A101), and A/California/07/2009 influenza virus hemagglutinin (pTEV-Cal09 HA-A101). mRNAs were transcribed to contain 101-nt-long poly(A) tails. One-methylpseudouridine (m1Ψ)-5′-triphosphate (TriLink) instead of uridine-5'-triphosphate (UTP) was used to generate modified nucleoside-containing mRNA. RNAs were capped using the m7G capping kit with 2′-*O*-methyltransferase (ScriptCap, CellScript) to obtain cap1. mRNA was purified by fast protein liquid chromatography (FPLC)

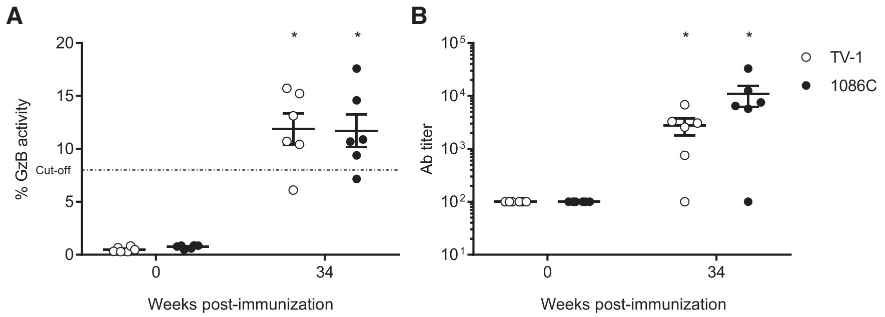

Figure 7. ADCC Activity after Nucleoside-Modified 1086C Env mRNA-LNP Immunization in Rhesus Macaques

ADCC activity in monkey sera obtained at weeks 0 and 34 was assessed. (A and B) ADCC responses against HIV-1 TV-1 and 1086C recombinant gp120-coated target cells (GTL assay). Granzyme B activity (A) and ADCC antibody titers (B) were determined. The cut-off for positivity (indicated with a horizontal dotted line) was ≥8% of granzyme B activity after subtracting the background measured in the uncoated cells. Antibody titers are expressed as the dilution at which the dilution curve interpolate with the 15% cut-off value. n = 6 monkeys and each symbol represents one animal. Means of values with SEM are displayed. Statistical significance of differences (denoted by asterisk) was determined using two-way ANOVA with Bonferroni multiple comparisons (after log transformation of values in the case of B); $p < 0.01$.

(Akta Purifier, GE Healthcare), as described.[52] All mRNAs were analyzed by denaturing or native agarose gel electrophoresis and were stored frozen at −20°C.

LNP Formulation of the mRNA

FPLC-purified m1Ψ-containing firefly luciferase, HIV-1 Env, influenza virus hemagglutinin-encoding mRNAs, and polycytidylic acid (poly(C)) RNA (Sigma) were encapsulated in LNPs, using a self-assembly process as previously described,[53] wherein an ethanolic lipid mixture of ionizable cationic lipid (proprietary to Acuitas Therapeutics), phosphatidylcholine, cholesterol, and polyethylene glycol (PEG)-lipid was rapidly mixed with an aqueous solution containing mRNA at acidic pH. The RNA-loaded particles were characterized and subsequently stored at −80°C at a concentration of 1 μg/μL. The mean hydrodynamic diameter of these mRNA-LNPs was ~80 nm, with a polydispersity index of 0.02–0.06 and an encapsulation efficiency of ~95% LNP.

Cell Culture

HEK293T cells (ATCC) were cultured in DMEM supplemented with 2 mM L-glutamine (Life Technologies) and 10% fetal calf serum (FCS) (HyClone) (complete medium). The 293T cell line was tested for mycoplasma contamination after receipt from ATCC and before expansion and cryopreservation.

The 293F cells (Life Technologies) were cultured in Freestyle 293 Expression Medium (Gibco). The 293F cell line was tested for mycoplasma contamination after receipt from Life Technologies and before expansion and cryopreservation.

mRNA Transfection

Transfection of HEK293T cells was performed with TransIT-mRNA (Mirus Bio), according to the manufacturer's instructions: mRNA (0.3 μg) was combined with TransIT-mRNA Reagent (0.34 μL) and Boost Reagent (0.22 μL) in 17 μL serum-free medium, and the complex was added to 5×10^4 cells in 183 μL complete medium. After overnight incubation at 37°C, supernatant was collected and cells were lysed for 1 h on ice in radio immunoprecipitation assay (RIPA) buffer (Sigma) at 18 h post-transfection.

2.5×10^5 HEK293F cells were transfected with 0.1 μg mRNA in 24-well plates using TransIT-mRNA, following the manufacturer's instructions. Cells were incubated at 37°C, harvested at 72 h, then washed with PBS with 1% BSA and resuspended at 2×10^7 cells/mL.

Western Blot Analysis of Env Protein Expression

Whole-cell lysates and supernatants from 1086C Env mRNA-transfected cells were assayed for Env protein by denaturing SDS-PAGE western blot. Samples were combined with 4× Laemmli buffer (Bio-Rad) and incubated at 95°C for 10 min, then separated on a 4%–15% precast polyacrylamide Mini-Protean TGX gel (Bio-Rad) for 1 h at 150 V. Transfer to polyvinylidene fluoride (PVDF) membrane was performed using a semi-dry apparatus (Ellard Instrumentation) at 10 V for 1 h. The membrane was blocked with 5% non-fat dry milk in Tris-buffered saline (TBS) buffer containing 0.1% Tween-20. Env protein was detected using 1:2,000 dilution of 1170[54] rabbit serum overnight at 4°C, followed by secondary donkey anti-rabbit horseradish peroxidase (HRP)-IgG (1:5,000; Jackson ImmunoResearch Laboratories) incubation for 1 h at room temperature. Blots were developed using Amersham ECL Western Blotting Detection Reagent on an Amersham Imager 600 (both from GE Healthcare) system.

Immunization of Rabbits and Monkeys

Rabbits

Female New Zealand white rabbits aged 6 weeks were obtained from Charles River Laboratories. Animals were induced with butorphanol (0.2 mg/kg) and dexmedetomidine (0.02 mg/kg) subcutaneously, masked with isoflurane, and then shaved on their backs and injected intradermally with mRNA-LNPs diluted in PBS. Six sites of injection (45 μL each) were used. Animals were randomly designated to experimental groups. After injections, the rabbits were reversed

with atipamezole (0.2 mg/kg) subcutaneously and monitored until fully recovered.

Monkeys

Ketamine-anesthetized animals were shaved on their backs and injected intradermally with mRNA-LNPs diluted in PBS. Ten sites of injection (60 μL each) were used. Animals of similar age and weight were randomly designated to dose groups.

Blood Collection

Rabbits

Blood was collected from the aural artery or the lateral saphenous vein. The animals were induced with the medications listed above and maintained on isoflurane anesthesia during blood collection. Blood was centrifuged for 10 min at 3,000 rpm in an Eppendorf microcentrifuge, and the serum was stored at −80°C and used for ELISA, ADCC, and virus neutralization assays.

Monkeys

Blood was collected by femoral or peripheral venipuncture under ketamine anesthesia. Serum separator tubes (SSTs) were centrifuged for 20 min at 1,750 rpm, and serum was collected and stored at −80°C for ELISA and neutralization analysis.

Antibody Reagents

The following antibodies were used for ELISAs: goat anti-rabbit IgG HRP conjugate (Millipore) and rabbit anti-monkey IgG HRP conjugate (Sigma) were used as detection antibodies for measuring anti-gp120 IgG titers.

The following antibodies and staining reagents were used for the *in vitro*-binding studies: goat anti-human IgG Fc-PE secondary antibody (Invitrogen); LIVE/DEAD Aqua Dead Cell Stain Kit (Life Technologies); CH65;[55] and HIV nAbs CH58,[14] CH106,[15] and 17b.[16]

Peptides and Proteins

The HIV-1 1086C gp120Δ7/293F/Mon was used as a coating antigen for ELISAs. The blocking protein for ELISAs was BSA (Sigma).

Indirect Fluorescence Flow Cytometry Analyses of mRNA-Transfected 293F Cells

4×10^5 transfected cells were incubated at 4°C for 1 h with 30 μg/mL nAb, then washed in PBS with 1% BSA. Cells were then incubated at 4°C for 30 min with goat anti-human IgG Fc-PE secondary antibody (2.5 μg/mL, Invitrogen). Finally, cells were incubated with a LIVE/DEAD differential stain (Life Technologies), following the manufacturer's instructions, and washed in PBS with 1% BSA. The presence of PE-labeled antibody on cell surfaces was detected on a BD LSRII flow cytometer. At least 10,000 events for each sample were recorded and data were analyzed with the FlowJo 10 software.

ELISA

HIV-1 gp120-specific IgG in rabbit and monkey sera was measured by ELISA. Immulon 4 HBX (rabbit samples) and Nunc MaxiSorp (monkey samples) high-binding plates were coated with 100 μL purified HIV-1 1086C gp120Δ7/293F/Mon gp120 at a final concentration of 1 μg/mL in PBS overnight at 4°C. The plates were washed three times with wash buffer (0.05% Tween-20 in PBS) and then blocked with blocking buffer (2% BSA in PBS) for 1 h at room temperature (RT), after which the plates were washed once with wash buffer. Dilutions of serum samples for the gp120-specific IgG measurement were made in blocking buffer and incubated on the plate (100 μL/well) for 1.5 h at RT. Samples were removed and the plate was washed four times with wash buffer. Detection antibodies were diluted 1:10,000 (rabbit) or 1:20,000 (monkey) in blocking buffer and incubated (100 μL/well) for 1 h. After four washes, 3,3',5,5'-Tetramethylbenzidine (TMB) substrate mixture (KPL) was added at 100 μL/well for 20 min. 2 N sulfuric acid (50 μL/well) was used to stop the reaction, and the optical density was read at 450 nm on a Dynex MRX Revelation microplate reader. Endpoint dilution titers were defined as the highest reciprocal dilution of serum to give an optical density (OD) greater than the sum of the background OD plus 0.01 units.

HIV-1 Virus Neutralization Assays

Neutralizing antibody activity was measured in 96-well culture plates by using Tat-regulated luciferase (Luc) reporter gene expression to quantify reductions in virus infection in TZM-bl cells. TZM-bl cells were obtained from the NIH AIDS Research and Reference Reagent Program, as contributed by John Kappes and Xiaoyun Wu. Assays were performed with HIV-1 Env-pseudotyped viruses as described previously.[17] Test samples were diluted over a range of 1:20 to 1:43,740 in cell culture medium and pre-incubated with virus (∼150,000 relative light unit equivalents) for 1 h at 37°C before the addition of cells. Following a 48-h incubation, cells were lysed and Luc activity was determined using a microtiter plate luminometer and BriteLite Plus Reagent (PerkinElmer). Neutralization titers are the serum dilution at which relative luminescence units (RLUs) were reduced by 50% compared to RLUs in virus control wells after subtraction of background RLUs in cell control wells. Serum samples were heat inactivated at 56°C for 1 h prior to assay.

ADCC Assays

Both the flow cytometry-based (GTL)[56] and the luciferase-based (Luc)[57] assays were utilized in this study. The subtype C HIV-1 1086C and TV-1 recombinant gp120 proteins were used to coat the cells in the GTL assay. The cut-off for positivity in the GTL assay was ≥8% of granzyme B activity after subtracting the background noted in the absence of any antibody (target and effector cells only). We also tested the samples using the luciferase-based (Luc) ADCC assay against the HIV-1 1086C transmitted/founder IMC-infected target cells. The IMC is ecto-IMCs generated using the NL4-3 HIV backbone with the insertion of the HIV-1 1086C Env and the luciferase reporter genes. The analysis of the results was conducted after subtracting the background detected with the pre-immunization (baseline) samples. After baseline subtraction, results were considered positive where the percent specific killing was ≥15%.

Statistical Analysis

Statistical analysis was performed using GraphPad Prism. Data were compared using one-way or two-way ANOVA corrected for multiple comparisons (Bonferroni method).

SUPPLEMENTAL INFORMATION

Supplemental Information can be found online at https://doi.org/10.1016/j.omtn.2019.03.003.

AUTHOR CONTRIBUTIONS

N.P., D.W., P.P., and S.-L.H. designed the vaccine studies. N.P., D.W.C., R.J.P., G.F., H.M., P.P., and I.T. performed the studies. D.C.M. and C.C.L. designed the HIV neutralization experiments. B.L.M., Y.K.T., C.J.B., T.D.M., and M.J.H. supplied reagents. N.P., D.W., B.F.H., and K.K. wrote the paper with help from the co-authors.

CONFLICTS OF INTEREST

In accordance with the University of Pennsylvania policies and procedures and our ethical obligations as researchers, we report that K.K. and D.W. are named on patents that describe the use of nucleoside-modified mRNA as a platform to deliver therapeutic proteins. D.W. and N.P. are also named on a patent describing the use of modified mRNA in lipid nanoparticles as a vaccine platform. We have disclosed those interests fully to the University of Pennsylvania, and we have in place an approved plan for managing any potential conflicts arising from the licensing of our patents.

ACKNOWLEDGMENTS

The HIV-1 Consensus C Env Peptide Set (9499) was obtained through the NIH AIDS Reagent Program, Division of AIDS, NIAID, NIH. We thank Drs. Robert Parks and Kevin O. Saunders for providing the HIV-1 1086C gp120Δ7/293F/Mon protein. We thank Dr. Robert W. Doms for providing rabbit serum 1170. We thank the Research Support Service at Washington National Primate Research Center (WaNPRC) for veterinarian care, Taryn Urion and Baoping Tian for technical assistance, Brad Cleveland for the preparation of inocula, and Deb Diamond for project management of the monkey studies. We thank Michael J. Hogan, for valuable feedback on the manuscript. The rhesus macaque study was supported in part by NIH grant P51 OD010425 to WaNPRC. D.W.C. was funded by UM1 AI100645 from the Duke CHAVI–Immunogen Discovery, Division of AIDS, NIAID, NIH. Flow cytometry was performed in the Duke Human Vaccine Institute Flow Cytometry Facility (Durham, NC). Neutralization assays were supported by NIH/NIAID contract HHSN272201100016C. R.J.P. and B.F.H. were supported by the Center for HIV/AIDS Vaccine Immunology-Immunogen Discovery (CHAVI-ID; UMI-AI100645) grant from NIH/NIAID/DAIDS. D.W. was supported by the NIAID of the NIH under award numbers R01-AI050484, R01-AI124429, and R01-AI084860 and by the Bill & Melinda Gates Foundation Collaboration for AIDS Vaccine Discovery (CAVD) 0PP1033102.

REFERENCES

1. Wei, X., Decker, J.M., Wang, S., Hui, H., Kappes, J.C., Wu, X., Salazar-Gonzalez, J.F., Salazar, M.G., Kilby, J.M., Saag, M.S., et al. (2003). Antibody neutralization and escape by HIV-1. Nature *422*, 307–312.
2. Sliepen, K., and Sanders, R.W. (2016). HIV-1 envelope glycoprotein immunogens to induce broadly neutralizing antibodies. Expert Rev. Vaccines *15*, 349–365.
3. McCoy, L.E., and Weiss, R.A. (2013). Neutralizing antibodies to HIV-1 induced by immunization. J. Exp. Med. *210*, 209–223.
4. Bahl, K., Senn, J.J., Yuzhakov, O., Bulychev, A., Brito, L.A., Hassett, K.J., Laska, M.E., Smith, M., Almarsson, Ö., Thompson, J., et al. (2017). Preclinical and Clinical Demonstration of Immunogenicity by mRNA Vaccines against H10N8 and H7N9 Influenza Viruses. Mol. Ther. *25*, 1316–1327.
5. Meyer, M., Huang, E., Yuzhakov, O., Ramanathan, P., Ciaramella, G., and Bukreyev, A. (2018). Modified mRNA-Based Vaccines Elicit Robust Immune Responses and Protect Guinea Pigs From Ebola Virus Disease. J. Infect. Dis. *217*, 451–455.
6. Pardi, N., Hogan, M.J., Naradikian, M.S., Parkhouse, K., Cain, D.W., Jones, L., Moody, M.A., Verkerke, H.P., Myles, A., Willis, E., et al. (2018). Nucleoside-modified mRNA vaccines induce potent T follicular helper and germinal center B cell responses. J. Exp. Med. *215*, 1571–1588.
7. Pardi, N., Hogan, M.J., Pelc, R.S., Muramatsu, H., Andersen, H., DeMaso, C.R., Dowd, K.A., Sutherland, L.L., Scearce, R.M., Parks, R., et al. (2017). Zika virus protection by a single low-dose nucleoside-modified mRNA vaccination. Nature *543*, 248–251.
8. Pardi, N., Parkhouse, K., Kirkpatrick, E., McMahon, M., Zost, S.J., Mui, B.L., Tam, Y.K., Karikó, K., Barbosa, C.J., Madden, T.D., et al. (2018). Nucleoside-modified mRNA immunization elicits influenza virus hemagglutinin stalk-specific antibodies. Nat. Commun. *9*, 3361.
9. Richner, J.M., Himansu, S., Dowd, K.A., Butler, S.L., Salazar, V., Fox, J.M., Julander, J.G., Tang, W.W., Shresta, S., Pierson, T.C., et al. (2017). Modified mRNA Vaccines Protect against Zika Virus Infection. Cell *168*, 1114–1125.e10.
10. Lindgren, G., Ols, S., Liang, F., Thompson, E.A., Lin, A., Hellgren, F., Bahl, K., John, S., Yuzhakov, O., Hassett, K.J., et al. (2017). Induction of Robust B Cell Responses after Influenza mRNA Vaccination Is Accompanied by Circulating Hemagglutinin-Specific ICOS+ PD-1+ CXCR3+ T Follicular Helper Cells. Front. Immunol. *8*, 1539.
11. Crotty, S. (2014). T follicular helper cell differentiation, function, and roles in disease. Immunity *41*, 529–542.
12. Havenar-Daughton, C., Lee, J.H., and Crotty, S. (2017). Tfh cells and HIV bnAbs, an immunodominance model of the HIV neutralizing antibody generation problem. Immunol. Rev. *275*, 49–61.
13. Keele, B.F., Giorgi, E.E., Salazar-Gonzalez, J.F., Decker, J.M., Pham, K.T., Salazar, M.G., Sun, C., Grayson, T., Wang, S., Li, H., et al. (2008). Identification and characterization of transmitted and early founder virus envelopes in primary HIV-1 infection. Proc. Natl. Acad. Sci. USA *105*, 7552–7557.
14. Liao, H.X., Bonsignori, M., Alam, S.M., McLellan, J.S., Tomaras, G.D., Moody, M.A., Kozink, D.M., Hwang, K.K., Chen, X., Tsao, C.Y., et al. (2013). Vaccine induction of antibodies against a structurally heterogeneous site of immune pressure within HIV-1 envelope protein variable regions 1 and 2. Immunity *38*, 176–186.
15. Liao, H.X., Lynch, R., Zhou, T., Gao, F., Alam, S.M., Boyd, S.D., Fire, A.Z., Roskin, K.M., Schramm, C.A., Zhang, Z., et al.; NISC Comparative Sequencing Program (2013). Co-evolution of a broadly neutralizing HIV-1 antibody and founder virus. Nature *496*, 469–476.
16. Sullivan, N., Sun, Y., Sattentau, Q., Thali, M., Wu, D., Denisova, G., Gershoni, J., Robinson, J., Moore, J., and Sodroski, J. (1998). CD4-Induced conformational changes in the human immunodeficiency virus type 1 gp120 glycoprotein: consequences for virus entry and neutralization. J. Virol. *72*, 4694–4703.
17. Montefiori, D.C. (2009). Measuring HIV neutralization in a luciferase reporter gene assay. Methods Mol. Biol. *485*, 395–405.
18. Yates, N.L., Liao, H.X., Fong, Y., deCamp, A., Vandergrift, N.A., Williams, W.T., Alam, S.M., Ferrari, G., Yang, Z.Y., Seaton, K.E., et al. (2014). Vaccine-induced

Env V1-V2 IgG3 correlates with lower HIV-1 infection risk and declines soon after vaccination. Sci. Transl. Med. *6*, 228ra39.

19. Balla-Jhagjhoorsingh, S.S., Corti, D., Heyndrickx, L., Willems, E., Vereecken, K., Davis, D., and Vanham, G. (2013). The N276 glycosylation site is required for HIV-1 neutralization by the CD4 binding site specific HJ16 monoclonal antibody. PLoS ONE *8*, e68863.
20. Falkowska, E., Le, K.M., Ramos, A., Doores, K.J., Lee, J.H., Blattner, C., Ramirez, A., Derking, R., van Gils, M.J., Liang, C.H., et al. (2014). Broadly neutralizing HIV antibodies define a glycan-dependent epitope on the prefusion conformation of gp41 on cleaved envelope trimers. Immunity *40*, 657–668.
21. Huang, J., Kang, B.H., Pancera, M., Lee, J.H., Tong, T., Feng, Y., Imamichi, H., Georgiev, I.S., Chuang, G.Y., Druz, A., et al. (2014). Broad and potent HIV-1 neutralization by a human antibody that binds the gp41-gp120 interface. Nature *515*, 138–142.
22. Li, Y., O'Dell, S., Walker, L.M., Wu, X., Guenaga, J., Feng, Y., Schmidt, S.D., McKee, K., Louder, M.K., Ledgerwood, J.E., et al. (2011). Mechanism of neutralization by the broadly neutralizing HIV-1 monoclonal antibody VRC01. J. Virol. *85*, 8954–8967.
23. Sok, D., Doores, K.J., Briney, B., Le, K.M., Saye-Francisco, K.L., Ramos, A., Kulp, D.W., Julien, J.P., Menis, S., Wickramasinghe, L., et al. (2014). Promiscuous glycan site recognition by antibodies to the high-mannose patch of gp120 broadens neutralization of HIV. Sci. Transl. Med. *6*, 236ra63.
24. Trkola, A., Purtscher, M., Muster, T., Ballaun, C., Buchacher, A., Sullivan, N., Srinivasan, K., Sodroski, J., Moore, J.P., and Katinger, H. (1996). Human monoclonal antibody 2G12 defines a distinctive neutralization epitope on the gp120 glycoprotein of human immunodeficiency virus type 1. J. Virol. *70*, 1100–1108.
25. McCoy, L.E., van Gils, M.J., Ozorowski, G., Messmer, T., Briney, B., Voss, J.E., Kulp, D.W., Macauley, M.S., Sok, D., Pauthner, M., et al. (2016). Holes in the Glycan Shield of the Native HIV Envelope Are a Target of Trimer-Elicited Neutralizing Antibodies. Cell Rep. *16*, 2327–2338.
26. Klasse, P.J., Ketas, T.J., Cottrell, C.A., Ozorowski, G., Debnath, G., Camara, D., Francomano, E., Pugach, P., Ringe, R.P., LaBranche, C.C., et al. (2018). Epitopes for neutralizing antibodies induced by HIV-1 envelope glycoprotein BG505 SOSIP trimers in rabbits and macaques. PLoS Pathog. *14*, e1006913.
27. Landais, E., Huang, X., Havenar-Daughton, C., Murrell, B., Price, M.A., Wickramasinghe, L., Ramos, A., Bian, C.B., Simek, M., Allen, S., et al. (2016). Broadly Neutralizing Antibody Responses in a Large Longitudinal Sub-Saharan HIV Primary Infection Cohort. PLoS Pathog. *12*, e1005369.
28. Zhou, T., Georgiev, I., Wu, X., Yang, Z.Y., Dai, K., Finzi, A., Kwon, Y.D., Scheid, J.F., Shi, W., Xu, L., et al. (2010). Structural basis for broad and potent neutralization of HIV-1 by antibody VRC01. Science *329*, 811–817.
29. Walker, L.M., Huber, M., Doores, K.J., Falkowska, E., Pejchal, R., Julien, J.P., Wang, S.K., Ramos, A., Chan-Hui, P.Y., Moyle, M., et al.; Protocol G Principal Investigators (2011). Broad neutralization coverage of HIV by multiple highly potent antibodies. Nature *477*, 466–470.
30. Sok, D., Laserson, U., Laserson, J., Liu, Y., Vigneault, F., Julien, J.P., Briney, B., Ramos, A., Saye, K.F., Le, K., et al. (2013). The effects of somatic hypermutation on neutralization and binding in the PGT121 family of broadly neutralizing HIV antibodies. PLoS Pathog. *9*, e1003754.
31. Mouquet, H., Scheid, J.F., Zoller, M.J., Krogsgaard, M., Ott, R.G., Shukair, S., Artyomov, M.N., Pietzsch, J., Connors, M., Pereyra, F., et al. (2010). Polyreactivity increases the apparent affinity of anti-HIV antibodies by heteroligation. Nature *467*, 591–595.
32. Klein, F., Mouquet, H., Dosenovic, P., Scheid, J.F., Scharf, L., and Nussenzweig, M.C. (2013). Antibodies in HIV-1 vaccine development and therapy. Science *341*, 1199–1204.
33. Andrabi, R., Voss, J.E., Liang, C.H., Briney, B., McCoy, L.E., Wu, C.Y., Wong, C.H., Poignard, P., and Burton, D.R. (2015). Identification of Common Features in Prototype Broadly Neutralizing Antibodies to HIV Envelope V2 Apex to Facilitate Vaccine Design. Immunity *43*, 959–973.
34. Yamamoto, T., Lynch, R.M., Gautam, R., Matus-Nicodemos, R., Schmidt, S.D., Boswell, K.L., Darko, S., Wong, P., Sheng, Z., Petrovas, C., et al. (2015). Quality and quantity of TFH cells are critical for broad antibody development in SHIVAD8 infection. Sci. Transl. Med. *7*, 298ra120.
35. Petrovas, C., Yamamoto, T., Gerner, M.Y., Boswell, K.L., Wloka, K., Smith, E.C., Ambrozak, D.R., Sandler, N.G., Timmer, K.J., Sun, X., et al. (2012). CD4 T follicular helper cell dynamics during SIV infection. J. Clin. Invest. *122*, 3281–3294.
36. Hong, J.J., Amancha, P.K., Rogers, K.A., Courtney, C.L., Havenar-Daughton, C., Crotty, S., Ansari, A.A., and Villinger, F. (2014). Early lymphoid responses and germinal center formation correlate with lower viral load set points and better prognosis of simian immunodeficiency virus infection. J. Immunol. *193*, 797–806.
37. Chowdhury, A., Del Rio Estrada, P.M., Tharp, G.K., Trible, R.P., Amara, R.R., Chahroudi, A., Reyes-Teran, G., Bosinger, S.E., and Silvestri, G. (2015). Decreased T Follicular Regulatory Cell/T Follicular Helper Cell (TFH) in Simian Immunodeficiency Virus-Infected Rhesus Macaques May Contribute to Accumulation of TFH in Chronic Infection. J. Immunol. *195*, 3237–3247.
38. Blackburn, M.J., Zhong-Min, M., Caccuri, F., McKinnon, K., Schifanella, L., Guan, Y., Gorini, G., Venzon, D., Fenizia, C., Binello, N., et al. (2015). Regulatory and Helper Follicular T Cells and Antibody Avidity to Simian Immunodeficiency Virus Glycoprotein 120. J. Immunol. *195*, 3227–3236.
39. Bonsignori, M., Wiehe, K., Grimm, S.K., Lynch, R., Yang, G., Kozink, D.M., Perrin, F., Cooper, A.J., Hwang, K.K., Chen, X., et al. (2014). An autoreactive antibody from an SLE/HIV-1 individual broadly neutralizes HIV-1. J. Clin. Invest. *124*, 1835–1843.
40. Haynes, B.F., Fleming, J., St Clair, E.W., Katinger, H., Stiegler, G., Kunert, R., Robinson, J., Scearce, R.M., Plonk, K., Staats, H.F., et al. (2005). Cardiolipin polyspecific autoreactivity in two broadly neutralizing HIV-1 antibodies. Science *308*, 1906–1908.
41. Liu, M., Yang, G., Wiehe, K., Nicely, N.I., Vandergrift, N.A., Rountree, W., Bonsignori, M., Alam, S.M., Gao, J., Haynes, B.F., and Kelsoe, G. (2015). Polyreactivity and autoreactivity among HIV-1 antibodies. J. Virol. *89*, 784–798.
42. Behrens, A.J., Kumar, A., Medina-Ramirez, M., Cupo, A., Marshall, K., Cruz Portillo, V.M., Harvey, D.J., Ozorowski, G., Zitzmann, N., Wilson, I.A., et al. (2018). Integrity of Glycosylation Processing of a Glycan-Depleted Trimeric HIV-1 Immunogen Targeting Key B-Cell Lineages. J. Proteome Res. *17*, 987–999.
43. Behrens, A.J., Vasiljevic, S., Pritchard, L.K., Harvey, D.J., Andev, R.S., Krumm, S.A., Struwe, W.B., Cupo, A., Kumar, A., Zitzmann, N., et al. (2016). Composition and Antigenic Effects of Individual Glycan Sites of a Trimeric HIV-1 Envelope Glycoprotein. Cell Rep. *14*, 2695–2706.
44. Chen, J., Kovacs, J.M., Peng, H., Rits-Volloch, S., Lu, J., Park, D., Zablowsky, E., Seaman, M.S., and Chen, B. (2015). HIV-1 ENVELOPE. Effect of the cytoplasmic domain on antigenic characteristics of HIV-1 envelope glycoprotein. Science *349*, 191–195.
45. Nguyen, H.T., Madani, N., Ding, H., Elder, E., Princiotto, A., Gu, C., Darby, P., Alin, J., Herschhorn, A., Kappes, J.C., et al. (2017). Evaluation of the contribution of the transmembrane region to the ectodomain conformation of the human immunodeficiency virus (HIV-1) envelope glycoprotein. Virol. J. *14*, 33.
46. Sanders, R.W., and Moore, J.P. (2017). Native-like Env trimers as a platform for HIV-1 vaccine design. Immunol. Rev. *275*, 161–182.
47. Pacheco, B., Alsahafi, N., Debbeche, O., Prévost, J., Ding, S., Chapleau, J.P., Herschhorn, A., Madani, N., Princiotto, A., Melillo, B., et al. (2017). Residues in the gp41 Ectodomain Regulate HIV-1 Envelope Glycoprotein Conformational Transitions Induced by gp120-Directed Inhibitors. J. Virol. *91*, e02219-16.
48. Kwong, P.D., and Mascola, J.R. (2018). HIV-1 Vaccines Based on Antibody Identification, B Cell Ontogeny, and Epitope Structure. Immunity *48*, 855–871.
49. Chen, Y., Wang, S., and Lu, S. (2014). DNA Immunization for HIV Vaccine Development. Vaccines (Basel) *2*, 138–159.
50. Pardi, N., Muramatsu, H., Weissman, D., and Karikó, K. (2013). In vitro transcription of long RNA containing modified nucleosides. Methods Mol. Biol. *969*, 29–42.
51. Thess, A., Grund, S., Mui, B.L., Hope, M.J., Baumhof, P., Fotin-Mleczek, M., and Schlake, T. (2015). Sequence-engineered mRNA Without Chemical Nucleoside Modifications Enables an Effective Protein Therapy in Large Animals. Mol. Ther. *23*, 1456–1464.
52. Weissman, D., Pardi, N., Muramatsu, H., and Karikó, K. (2013). HPLC purification of in vitro transcribed long RNA. Methods Mol. Biol. *969*, 43–54.

53. Pardi, N., Tuyishime, S., Muramatsu, H., Kariko, K., Mui, B.L., Tam, Y.K., Madden, T.D., Hope, M.J., and Weissman, D. (2015). Expression kinetics of nucleoside-modified mRNA delivered in lipid nanoparticles to mice by various routes. J. Control. Release *217*, 345–351.

54. Hoffman, T.L., LaBranche, C.C., Zhang, W., Canziani, G., Robinson, J., Chaiken, I., Hoxie, J.A., and Doms, R.W. (1999). Stable exposure of the coreceptor-binding site in a CD4-independent HIV-1 envelope protein. Proc. Natl. Acad. Sci. USA *96*, 6359–6364.

55. Whittle, J.R., Zhang, R., Khurana, S., King, L.R., Manischewitz, J., Golding, H., Dormitzer, P.R., Haynes, B.F., Walter, E.B., Moody, M.A., et al. (2011). Broadly neutralizing human antibody that recognizes the receptor-binding pocket of influenza virus hemagglutinin. Proc. Natl. Acad. Sci. USA *108*, 14216–14221.

56. Pollara, J., Hart, L., Brewer, F., Pickeral, J., Packard, B.Z., Hoxie, J.A., Komoriya, A., Ochsenbauer, C., Kappes, J.C., Roederer, M., et al. (2011). High-throughput quantitative analysis of HIV-1 and SIV-specific ADCC-mediating antibody responses. Cytometry A *79*, 603–612.

57. Pollara, J., Bonsignori, M., Moody, M.A., Liu, P., Alam, S.M., Hwang, K.K., Gurley, T.C., Kozink, D.M., Armand, L.C., Marshall, D.J., et al. (2014). HIV-1 vaccine-induced C1 and V2 Env-specific antibodies synergize for increased antiviral activities. J. Virol. *88*, 7715–7726.

OMTN, Volume *15*

Supplemental Information

Characterization of HIV-1 Nucleoside-Modified mRNA Vaccines in Rabbits and Rhesus Macaques

Norbert Pardi, Celia C. LaBranche, Guido Ferrari, Derek W. Cain, István Tombácz, Robert J. Parks, Hiromi Muramatsu, Barbara L. Mui, Ying K. Tam, Katalin Karikó, Patricia Polacino, Christopher J. Barbosa, Thomas D. Madden, Michael J. Hope, Barton F. Haynes, David C. Montefiori, Shiu-Lok Hu, and Drew Weissman

SUPPLEMENTAL FIGURES AND TABLES

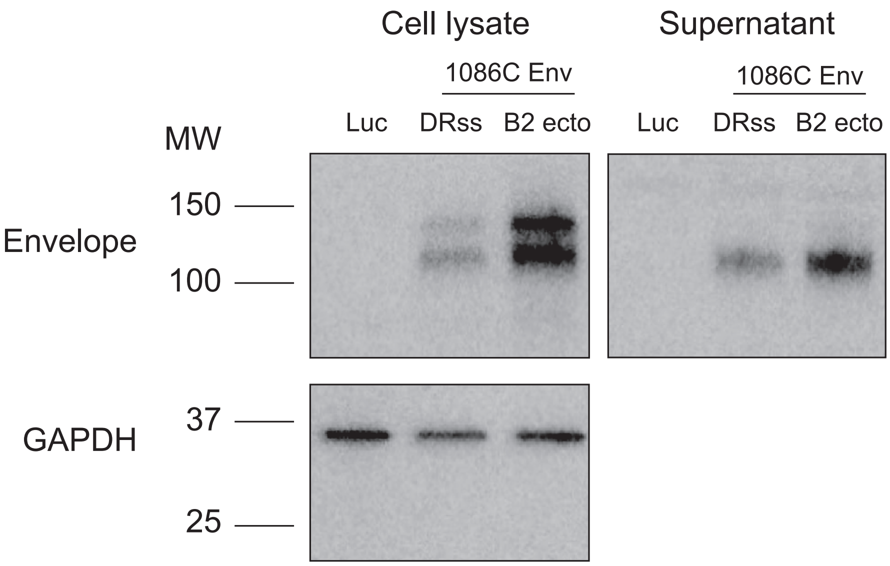

Figure S1. Protein production from HIV-1 1086C Env-encoding mRNAs in vitro
mRNAs were transfected into HEK293T cells. Env protein expression in cell lysate and supernatant was probed by Western blot, using firefly luciferase (Luc)-encoding mRNA-transfected cells as a negative control.

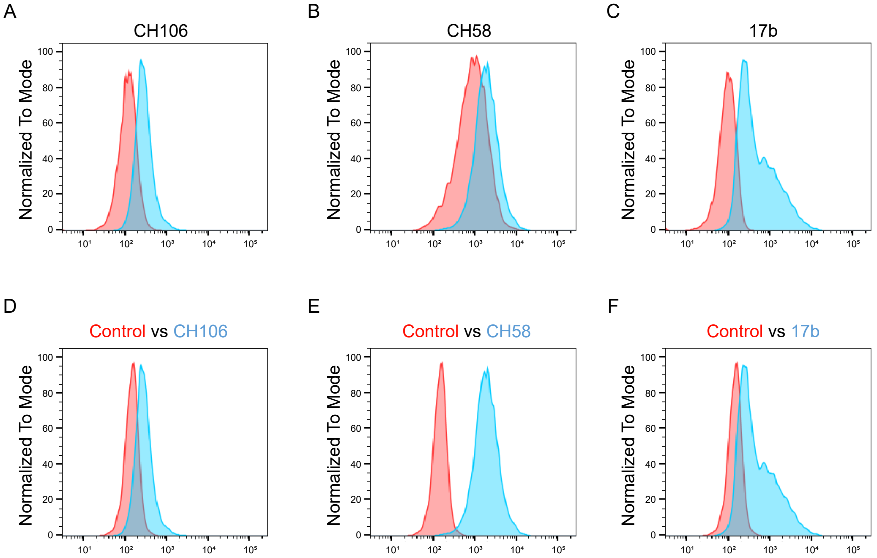

Figure S2. HIV nAb binding to 1086C B2 Ecto gp160 mRNA-transfected 293F cells
mRNAs were transfected into 293F cells. Panels A, B and C represent positive binding of three HIV gp120 antibodies to gp160 transfected cells (blue) relative to firefly luciferase transfected cells (red). Panels D, E and F show binding of the same three antibodies (blue) relative to an influenza virus anti-hemagglutinin negative control antibody, CH65 (red). Two independent experiments were performed with similar results.

ID	Sex	DOB	Age (years) Week 0	Weight (kg) Week 0
A16331	Male	5/8/2013	3.81	4.07
A16332	Male	5/25/2013	3.77	4.48
A16333	Male	6/10/2013	3.72	4.67
A16334	Male	5/18/2013	3.79	3.83
A16335	Male	7/21/2013	3.61	6.48
A16337	Male	9/28/2013	3.42	4.20

Table S1. Characteristics of rhesus macaques in vaccination experiments. Animal identification (ID) numbers, weight at the time of the first immunization, sex, and date of birth (DOB) are shown.
